# Association between High On-Aspirin Platelet Reactivity and Reduced Superoxide Dismutase Activity in Patients Affected by Type 2 Diabetes Mellitus or Primary Hypercholesterolemia

**DOI:** 10.3390/ijms21144983

**Published:** 2020-07-15

**Authors:** Cristina Barale, Franco Cavalot, Chiara Frascaroli, Katia Bonomo, Alessandro Morotti, Angelo Guerrasio, Isabella Russo

**Affiliations:** 1 Department of Clinical and Biological Sciences of Turin University, 10043 Orbassano, Turin, Italy; cristina.barale@unito.it (C.B.); alessandro.morotti@unito.it (A.M.); angelo.guerrasio@unito.it (A.G.); 2 Metabolic Disease and Diabetes Unit, San Luigi Gonzaga Hospital, 10043 Orbassano, Turin, Italy; fcavalot@fastwebnet.it (F.C.); chiarafrascaroli@gmail.com (C.F.); ka.bonomo@tiscali.it (K.B.)

**Keywords:** platelets, oxidative stress, superoxide dismutase, aspirin, thromboxane, platelet function analyzer-100

## Abstract

Platelet hyperactivation is involved in the established prothrombotic condition of metabolic diseases such as Type 2 Diabetes Mellitus (T2DM) and familial hypercholesterolemia (HC), justifying the therapy with aspirin, a suppressor of thromboxane synthesis through the irreversible inhibition of cyclooxygenase-1 (COX-1), to prevent cardiovascular diseases. However, some patients on aspirin show a higher than expected platelet reactivity due, at least in part, to a pro-oxidant milieu. The aim of this study was to investigate platelet reactivity in T2DM (*n* = 103) or HC (*n* = 61) patients (aspirin, 100 mg/day) and its correlation with biomarkers of redox function including the superoxide anion scavenger superoxide dismutase (SOD) and the in vivo marker of oxidative stress urinary 8-iso-prostaglandin F_2α_. As results, in T2DM and HC subjects the prevalence of high on-aspirin platelet reactivity was comparable when both non-COX-1-dependent and COX-1-dependent assays were performed, and platelet reactivity is associated with a lower SOD activity that in a stepwise linear regression appears as the only predictor of platelet reactivity. To conclude, in T2DM and HC, similarly, the impairment of redox equilibrium associated with a decrease of SOD activity could contribute to a suboptimal response to aspirin.

## 1. Introduction

Atherothrombosis is the primary cause of cardio- and cerebrovascular events in patients with Type 2 Diabetes Mellitus (T2DM) and platelet hyperactivation plays a pivotal role in the initiation and progression of thrombotic complications that follow the atherosclerotic plaque disruption [1,2,3].

Platelet activation is a multifactorial process where the platelet-platelet interaction provides a further source of intracellular signaling associated with the release of pro-inflammatory and pro-aggregating molecules, thus increasing the prothrombotic tendency.

Since the inhibition of platelet activation pathways with antiplatelet therapy is crucial for the prevention of atherothrombotic diseases, aspirin (acetylsalicylic acid) has been extensively used as medication in the prevention of cardiovascular events in T2DM patients due to its main ability to suppress thromboxane A_2_ (TXA_2_) synthesis, a powerful activator of platelet response and thrombus formation, by irreversibly inhibiting cyclooxygenase-1 (COX-1) activity [4,5].

However, in spite of its wide acceptance and utilization, the clinical aspirin effectiveness in protecting from vascular events has been questioned because of the occurrence of cerebro- and cardiovascular events even in the presence of low-dose aspirin treatment both in primary [4] and secondary prevention [5]. The role of biochemical and clinical features involved in the reduced aspirin effect in T2DM is a still debated issue—it has been shown that in T2DM patients, either genetic factors or secondary causes (metabolic disorders and/or inflammatory states) may be important contributors to the diminished aspirin action [6], demonstrating that the benefits of aspirin treatment may be overcome by aspirin-insensitive mechanisms of platelet activation [7,8].

In all cases, as a result of the insufficient inhibition of COX-1 activity by aspirin, a larger amount of both serum and urinary excretion of TXA_2_ metabolites has been found and associated with increased incidence of major adverse cardiovascular events [9,10,11,12].

Metabolic derangement in diabetes can account for the impairment of both endothelial and platelet function with the expression and release of mediators involved in the atherogenic process such as chemokines, adhesion molecules, and cytokines. Both acute and chronic hyperglycemia are associated with platelet activation in T2DM [13] and, also, the urinary excretion rate of 11-dehydro- TXB_2_ (11-dhTXB_2_) and 8-iso-prostaglandin F_2α_ (8-iso-PGF_2α_), a reliable marker of oxidative stress, correlate directly with metabolic control, suggesting a link between glycemic control, oxidative stress and platelet activation [14], with the caution that urine 11-dhTXB_2_ is not only a marker of platelet activity [15] but also a marker of inflammation, since it originates from other blood cells [16].

In normal conditions, a protective role in modulating redox status is exerted by superoxide dismutase (SOD), a class of enzymes that catalyze the dismutation of the superoxide anion into oxygen and hydrogen peroxide [17] and the extracellular SOD (EC-SOD or SOD3) is a major plasma extracellular antioxidant enzyme [18].

Under oxidative stress conditions, both arachidonic acid (AA) and circulating low-density lipoprotein (LDL), through a nonenzymatic process of peroxidation, increase the production of some biologically active prostanoids able to influence platelet function [19]. In particular, abnormal 8-iso-PGF_2α_ production has been found to correlate with soluble CD40 Ligand (sCD40L) biosynthesis [20], a marker of in vivo platelet activation, and is considered a crucial factor able to trigger a cascade of activation pathways substantially contributing to increased platelet responsiveness to common agonists.

The increased oxidative stress and endothelial activation related to glucose excursions are strictly associated with platelet hyperactivation [21,22,23].

Hypercholesterolemia (HC) per se predisposes to atherothrombotic complications through different mechanisms including platelet hyperactivity [24] and a reduced cardioprotective action of aspirin [25]. In this context, the impaired platelet metabolism and intraplatelet signaling pathways as a result of the influence of the concomitant metabolic and proatherogenic abnormalities may significantly contribute to increase platelet reactivity [26] and reduce the inhibitory effects of aspirin.

The aim of this study was to establish in T2DM or primary HC the relationships between a pattern of pro- and antioxidant, inflammation and endothelial dysfunction biomarkers and the presence of high on-aspirin residual platelet reactivity.

## 2. Results

Taking into account that aspirin action specifically inhibits COX-1 activity and that the serum TXB_2_ measurement represents the most accurate COX-1-dependent test, we focused this study on patients with serum TXB_2_ concentrations lower than 10 ng/mL, corresponding to more than 95% inhibition of COX-1 activity [27]. We excluded four out of 65 HC and 10 out of 113 T2DM patients from the final analysis since they showed serum TXB_2_ concentrations higher than 10 ng/mL, thus indicating patients with true resistance to aspirin, poor patient compliance or inability of once-daily dose of aspirin to inhibit newly formed platelets due to increased platelet turnover.

Main clinical and laboratory characteristics of the investigated subjects are summarized in Table 1.

In comparison with HC, T2DM patients showed significantly higher values of body mass index (BMI) (*p* = 0.008), glycosylated hemoglobin (HbA1c) (*p* < 0.0001), fasting blood glucose (*p* < 0.0001), and systolic blood pressure (*p* = 0.013) and lower levels of total cholesterol (TC) (*p* = 0.005), LDL-C (*p* = 0.019), and high-density lipoprotein cholesterol (HDL-C) (*p* < 0.0001).

### 2.1. Residual On-Aspirin Platelet Reactivity Assays in HC and T2DM Patients

**Figure 1:**
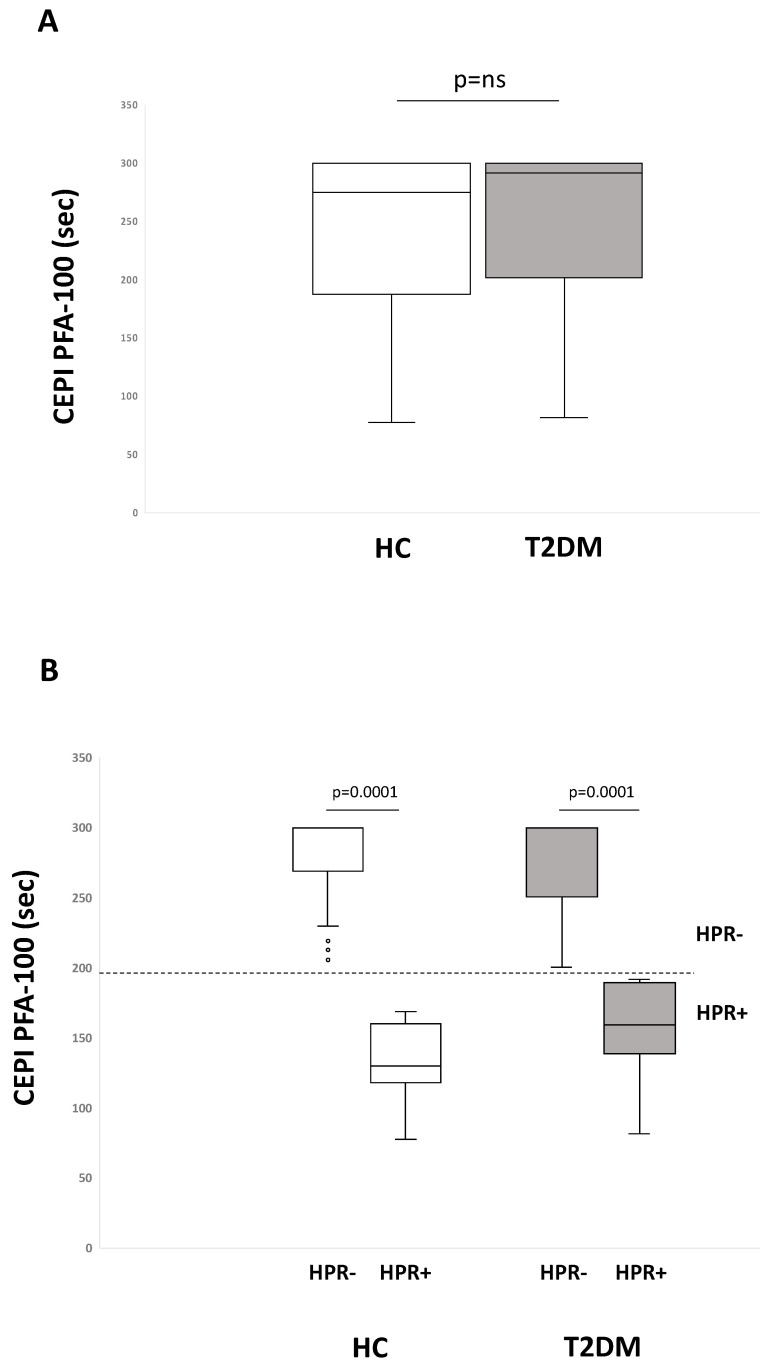
Box-plots showing collagen/epinephrine (CEPI) Platelet Function Analyzer-100 (PFA-100) closure time values in primary hypercholesterolemia (HC) and Type 2 Diabetes Mellitus (T2DM) patients (**A**) and in HC and T2DM patients stratified for the absence (-) or the presence (+) of high on-aspirin platelet reactivity (HPR) (**B**). Horizontal dotted line denotes cut-off value for residual platelet reactivity despite aspirin intake. Solid lines, median values; boxes, interquartile range; whiskers, nonoutlier range; open circles, outliers.

Aspirin intake, as expected, reduced platelet adhesion and aggregation under shear stress conditions, as mirrored by the prolongation of Platelet Function Analyzer (PFA)-100 closure times when collagen/epinephrine (CEPI) but not collagen/adenosine diphosphate (ADP) (CADP) PFA-100 cartridges were used. CEPI PFA-100 median values were 292 sec for T2DM and 275 sec for HC subjects (*p* = ns) (Figure 1A).

When stratified for the absence (HPR-) or the presence of high platelet reactivity (HPR+) based on the response to CEPI PFA-100 (cut-off 193 sec), the percentage of HPR+ in T2DM and HC subjects was similar (23% and 25%, respectively, *p* = ns) (Figure 1B) and within each group closure time values were significantly different between HPR+ and HPR- (*p* < 0.0001).

CADP PFA-100 closure time median values were 89 sec for HC and 85 sec for T2DM patients (*p* = ns) without significant differences between HPR- and HPR+ within each study group.

**Figure 2:**
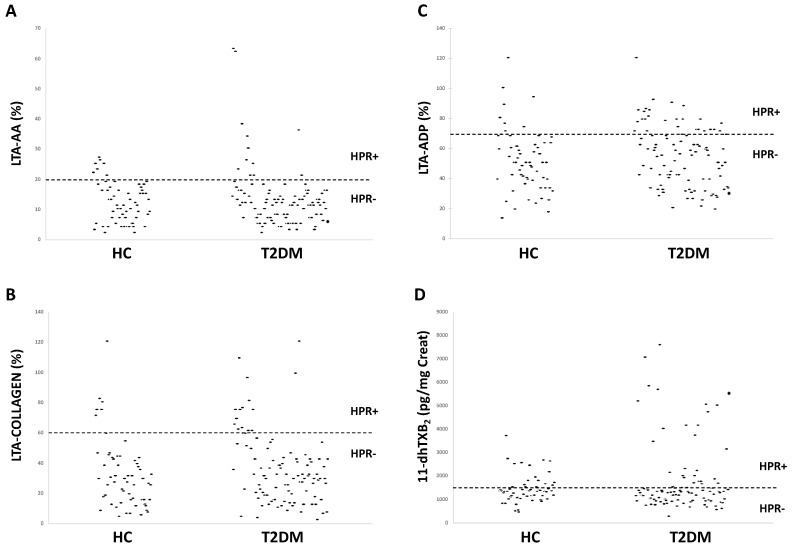
Distribution of light platelet aggregation (LTA) to arachidonic acid (AA)(**A**), collagen (**B**), adenosine diphosphate (ADP)(**C**) and of urinary 11-dehydro-thromboxane B_2_ (11-dhTXB_2_) levels (**D**) in primary hypercholesterolemia (HC) and Type 2 Diabetes Mellitus (T2DM) patients. Horizontal dotted line denotes, for each platelet function test, the cut-off value which separates subjects without (-) or with (+) high on-aspirin platelet reactivity (HPR).

Figure 2 shows in HC and T2DM patients the distribution of light transmission aggregation (LTA) tests in response to AA (Figure 2A), collagen (Figure 2B), and ADP (Figure 2C) with the dotted line which denotes the cut-off value for residual platelet reactivity despite aspirin intake and separates HPR+ (above the line) from HPR- (below the line) subjects. Accordingly with these limit values, in HC and T2DM subjects, the percentage of HPR+ was, respectively, 11% and 12% with LTA-AA (*p* = ns), 13% and 9% with LTA-collagen (*p* = ns), 13% and 24% with LTA-ADP (*p* = 0.01).

A persistent platelet activation was also evaluated by measuring the urinary 11-dhTXB_2_ (Figure 2D) given that 80% of urinary 11-dhTXB_2_ is platelet-derived [28]. Accordingly with a cut-off value of ≤ 1500 pg for 11-dhTXB_2_/mg creatinine corresponding to a good aspirin effect [29], the percentage of HPR+ was 25% in HC and 26% in T2DM patients (*p* = ns).

### 2.2. Metabolic Parameters and Biomarkers of Oxidative Stress, Inflammation, and Platelet Activation in HPR+ vs. HPR- according to CEPI PFA-100

We chose the response to CEPI PFA-100 as the parameter to distinguish HPR- from HPR+ and to compare them in terms of clinical and laboratory data within HC and T2DM patient groups. Indeed, among the platelet tests used here, PFA-100 is the most physiological since it was performed in whole blood and was potentially influenced by circulating biochemical factors, inflammatory and pro-oxidant molecules. Furthermore, it is rapid and easy to use, approved for measuring platelet dysfunction and widely used to detect aspirin response in clinical settings [30].

Whilst no significant difference was found between HPR+ and HPR- within HC subjects as far as the main clinical and metabolic parameters are concerned, T2DM HPR+ differed from HPR- for higher levels of TC (*p* = 0.005) and LDL-C (*p* = 0.013) (Table 2).

When oxidative stress, inflammation and platelet activation markers were investigated (Table 3), in comparison with HC, T2DM subjects showed significant higher circulating levels of—(i) the in vivo oxidative stress marker 8-iso-PGF_2α_ (*p* = 0.016), (ii) the in vivo platelet activation marker sCD40L (*p* < 0.0001), (iii) the subclinical inflammatory marker interleukin-6 (IL-6) (*p* = 0.0001).

Furthermore, T2DM patients showed higher levels of 11-dhTXB_2_ (*p* = 0.049) and lower concentrations of the antiatherogenic soluble RAGE (sRAGE) (*p* < 0.0001). A significant direct correlation was found between 8-iso-PGF_2α_ and fasting glucose (*r* = 0.250, *p* = 0.002) and HbA1c (*r* = 0.231, *p* = 0.004), thus indicating the effect of metabolic control on the increase of oxidative stress.

With regard to SOD activity, sCD40L, soluble E-selectin (sE-selectin) and soluble Intercellular Adhesion Molecule (sICAM) showed no statistically significant differences between HC and T2DM patients.

When subjects within each study population were stratified in HPR+ and HPR- according to their response to CEPI PFA-100 (Table 4), we found that HPR+ differed from HPR- for—(i) SOD activity (*p* = 0.002), soluble *p*-selectin (sP-selectin) (*p* = 0.001), sRAGE (*p* = 0.004), and 11-dhTXB_2_ (*p* = 0.035) levels in HC patients; (ii) SOD activity (*p* = 0.0001), 8-iso-PGF_2α_ (*p* < 0.0001), sCD40L (*p* = 0.003), and 11-dhTXB_2_ (*p* = 0.042) in T2DM patients.

### 2.3. Correlation Analyses

**Figure 3:**
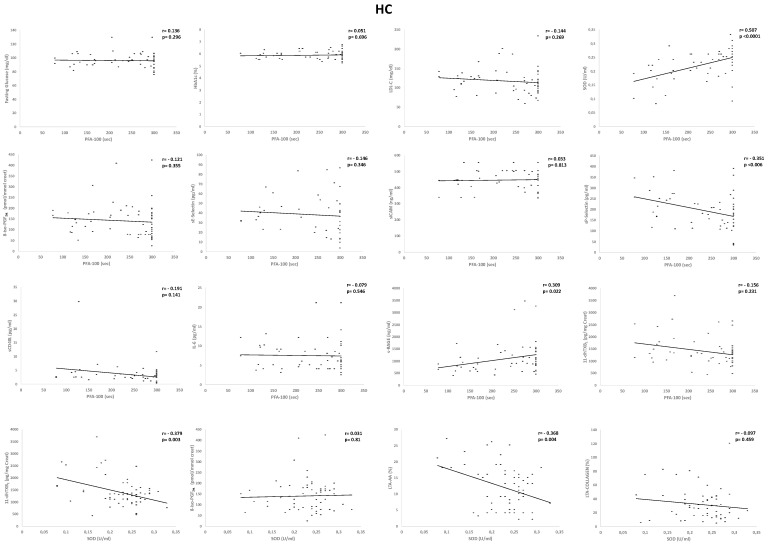
Univariate regression analysis of collagen/epinephrine (CEPI) Platelet Function Analyzer-100 (PFA-100) and superoxide dismutase (SOD) with biomarkers and platelet aggregation tests in subjects with primary hypercholesterolemia (HC). HbA1c, glycosylated hemoglobin; LDL-C, low-density lipoprotein cholesterol; 8-iso-PGF_2α_, 8-iso-prostaglandin F_2α_; IL-6, interleukin-6; 11-dhTXB_2_, 11-dehydro-thromboxane B_2_; LTA, light transmission aggregation.

In HC subjects—(i) CEPI PFA-100 closure times significantly correlated with SOD (*r* = 0.507, *p* < 0.0001), sRAGE (*r* = 0.309, *p* = 0.022), and sP-selectin (*r* = −0.351, *p* < 0.006), (ii) SOD inversely correlated with 11-dhTXB_2_ (*r* = −0.379, *p* = 0.003), and LTA-AA (*r* = 0.368, *p* = 0.004), (Figure 3).

**Figure 4:**
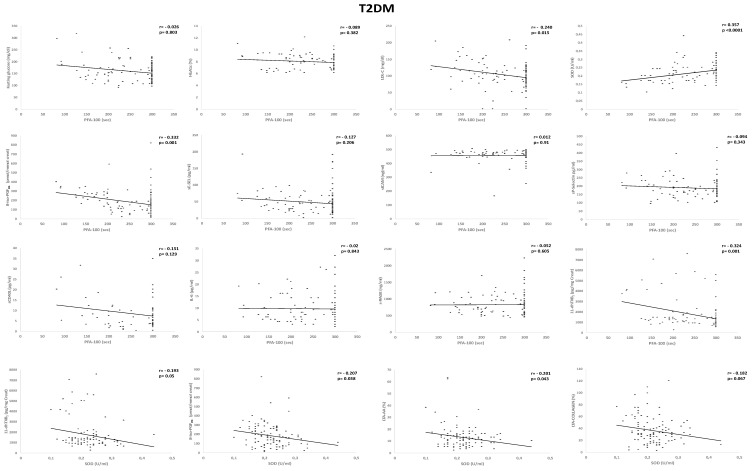
Univariate regression analysis of collagen/epinephrine (CEPI) Platelet Function Analyzer-100 (PFA-100) and superoxide dismutase (SOD) with biomarkers and platelet aggregation tests in subjects with Type 2 Diabetes Mellitus (T2DM). HbA1c, glycosylated hemoglobin; LDL-C, low-density lipoprotein cholesterol; 8-iso-PGF_2α_, 8-iso-prostaglandin F_2α_; IL-6, interleukin-6; 11-dhTXB_2_, 11-dehydro-thromboxane B_2_; LTA, light transmission aggregation.

In T2DM subjects—(i) CEPI PFA-100 closure times significantly correlated with LDL-C (*r* = −0.240, *p* = 0.015), SOD (*r* = 0.357, *p* < 0.0001), 11-dhTXB_2_ (*r* = −0.324, *p* = 0.001), and 8-iso-PGF_2α_ (*r* = −0.332, *p* = 0.001); (ii) SOD inversely correlated with 11-dhTXB_2_ (*r* = −0.195, *p* = 0.05), 8-iso-PGF_2α_ (*r* = −0.207, *p* = 0.038), LTA-AA (*r* = −0.201, *p* = 0.043), and LTA-collagen (*r* = −0.182, *p* = 0.067) (Figure 4).

Interestingly, a univariate regression analysis performed on the whole population showed that (i) SOD significantly correlated with LDL-C (*r* = −0.160, *p* = 0.0041), 8-iso-PGF_2α_ (*r* = −0.158, *p* = 0.05), 11-dhTXB_2_ (*r* = −0.213, *p* = 0.006). A stepwise regression analysis including clinical and laboratory data that differed between HC and T2DM patients and between HPR- and HPR+ within each group (BMI, HbA1c, fasting glucose, TC, HDL-C, LDL-C, SOD, sRAGE, 8-iso-PGF_2α_, IL-6, sCD40L), entered as independent variables, and CEPI PFA-100 as a dependent variable showed that only SOD significantly predicted platelet reactivity according to CEPI PFA-100 response in both HC (*t* = 5.941, *β* = 0.632, *p* < 0.0001) and T2DM (*t* = 2.873, *β* = 0.298, *p* = 0.005) patients.

## 3. Discussion

The main findings of this study are that—(i) T2DM and primary HC patients show comparable prevalence of high on-aspirin platelet reactivity when the non-COX-1-dependent CEPI PFA-100 and COX-1-dependent LTA-AA, LTA-collagen, urinary 11-dhTXB_2_ assays were performed; (ii) compared to HPR-, HPR+ subjects with T2DM or HC showed an imbalance of redox status corresponding to a lower activity of the oxidant scavenger SOD; (iii) SOD positively correlates with CEPI PFA-100 closure times and a stepwise linear regression yielded a model in which only SOD predicts platelet reactivity in both T2DM and HC.

Although an impaired aspirin ability to inhibit platelet aggregation has been frequently observed in T2DM if compared with the general population [31,32], in this study we show that T2DM and primary HC patients, all on 100 mg/d aspirin treatment, display a similar prevalence for HPR+ in most of the in vitro assays used to test platelet sensitivity to the inhibitory effects of aspirin. Notably, we excluded from the investigation subjects with serum TXB_2_ levels >10 ng/mL that mirror the failure of aspirin to effectively inhibit COX-1 activity for true “aspirin resistance”, poor compliance or insufficient aspirin dose effectiveness when platelet turnover is increased [33]; instead, we focused our investigations on platelets in which COX-1 is sufficiently inhibited by aspirin and the platelet reactivity is not strictly COX-1-dependent. After that, serum TXB_2_ concentrations did not differ between T2DM and HC patients unlike urinary 11-dhTXB_2_ levels, which provide a reliable information on the systemic rate of in vivo TXA_2_ biosynthesis derived from platelets and inflammatory cells through COX-2-dependent mechanisms, were higher in T2DM. Interestingly, our T2DM patients also showed higher levels of sCD40L and 8-iso-PGF_2α_ and the positive correlation between sCD40L and 11-dhTXB_2_ and 8-iso-PGF_2α_ (data not shown) confirms that CD40L release occurs during TXA_2_-dependent activation and is involved in the increased oxidant stress and lipid peroxidation in T2DM [20], respectively. The significant direct correlation between 8-iso-PGF_2α_ and HbA1c [19] may explain, at least in part, why T2DM patients show greater levels of 8-iso-PGF_2α_than HC, despite not explaining why 8-iso-PGF_2α_ but not HbA1c values, significantly differed between HPR+ and HPR- in T2DM. Of note, increased levels of sCD40L and 11-dhTXB_2_ characterized HPR+ in both T2DM and HC suggesting common biochemical mechanisms linking residual platelet reactivity and inflammation. We also observed that T2DM compared to HC subjects are also characterized by lower circulating levels of sRAGE, which is known to act as a protective factor against the deleterious effects of inflammation and oxidative stress [34]. This inflammatory environment also promotes lipid peroxidation with consequent activation of platelets and furthers oxidative stress, which could contribute to enhanced urinary excretion of 8-iso-PGF_2α_ more in T2DM than in HC subjects [35,36,37]. In light of this, it cannot be excluded that the lower sRAGE levels found in HPR+ subjects with HC may have a role in the higher plasma concentrations of sP-selectin observed in the same subjects.

We observed that the prevalence of HPR+ subjects in T2DM and HC is comparable when platelet reactivity was evaluated by CEPI PFA-100, LTA-AA, LTA-collagen, and 11-dhTXB_2_ levels, whereas a higher percentage of HPR+ subjects resulted in T2DM than in HC subjects when LTA-ADP was performed. Indeed, LTA-ADP, even if used to measure platelet sensitivity to aspirin, is not specific at measuring COX-1 activity and shows low agreement with LTA-AA and serum TXB_2_ [38].

The heterogeneity in the response and the different incidence of HPR+ among these tests are not surprising and confirm the clear difference in the ability of each of these tests to detect the on-aspirin treatment residual platelet reactivity [38]. Among them, LTA-AA is the COX-1-dependent test that better agrees with serum TXB_2_ because AA agonist exploits the specific pathway affected by aspirin and it is still considered the gold standard of platelet function. However, other mechanisms of platelet activation are highly likely to occur within whole blood in which COX-1 inhibition by aspirin can be bypassed. CEPI PFA-100, a non-COX-1-dependent test carried out in whole blood, is widely available, rapid and simple to use even though it tends to overestimate the prevalence of high residual platelet reactivity [39]. Nevertheless, lower CEPI PFA-100 closure times denoting platelet hyporesponsiveness to aspirin are clinically associated with increased vascular events [40]. In light of this, we used the response to CEPI PFA-100 as the phenotypic identifier for comparison of clinical and laboratory data between HPR- and HPR+ subjects within T2DM and HC patient groups.

To the best of our knowledge, we show for the first time that a high on-aspirin residual platelet reactivity based on CEPI PFA-100 method in both T2DM and HC patients is associated with a reduced activity of the extracellular SOD, a major extracellular antioxidant enzyme deeply involved in modulating the cell redox status and highly expressed in blood vessels, particularly in arterial walls where it represents up to 70% of the total SOD activity [41]. Indeed, differently from reactive oxygen species (ROS)-generating enzymes, conflicting results came from human studies that evaluated the relationship between diseases and antioxidant enzymes given that for ROS scavenger enzymes, decreased or increased activities (or levels) have been reported [42,43,44,45,46].

It is important to underline that, by catalyzing the dismutation of superoxide into oxygen and hydrogen peroxide, SOD can acts as a pro-oxidant, thus other antioxidant enzymes, such as catalase and glutathione peroxidase, are normally needed to avoid a redox imbalance with consequent dangerous effects due to increased oxidant milieu [47]. Oxidative stress plays a major role in the development of vascular complications in different clinical settings and the extracellular SOD exerts vascular protection against oxidant species [41]. Interestingly, in our study, in both T2DM and HC, SOD activity is lower in HPR+ than in HPR- subjects and positively correlates with CEPI PFA-100 closure times, thus suggesting the implication of this antioxidant enzyme in influencing platelet function. Furthermore, a negative correlation was found between SOD and 11-dhTXB_2_ in HC, between SOD and 11-dhTXB_2_ and 8-iso-PGF_2α_in T2DM. The relationship between SOD and 11-dhTXB_2_ is likely to be attributable to the role of oxidant species, such as hydrogen peroxide, which are a stimulus for the production of platelet thromboxane A_2_ [48], thus justifying, at least in part, the finding that animals deficient in antioxidants showed enhanced platelet activation, platelet-rich thrombi, and occluded vessels to a greater extent than wild-type ones [49].

As known, metabolic diseases such as T2DM and HC are associated with an oxidative stress milieu as a consequence of intracellular hyperglycemia or increased oxidation of fatty acids [50] and superoxide, in particular, is the initial oxygen free radical formed by the mitochondria, which is then converted to other more reactive species that can damage cells [51]. It is known that when ROS generation exceeds the capacity of the antioxidant defense systems and/or in the presence of a reduction of antioxidant enzymes, oxidative stress occurs and represents the primary cause of endothelial dysfunction and vascular damage in metabolic diseases [52]. A large body of evidence supports the close link between increased oxidative stress, which characterizes both T2DM and HC, and a less-than-expected response to aspirin, with different mechanisms [53]. Actually, chronic oxidative stress [54] and decreased antioxidant capacity [55] have been documented in diabetes but, for the first time, we show plasma SOD as a contributor to promote redox status imbalance and it appears as the only predictor of CEPI PFA-100 in on-aspirin-treated T2DM and primary HC patients. Of course, further studies to fully elucidate in these subjects not only the radical scavenging enzyme activity but also the plasma nonenzymatic antioxidant capacity would be required to better define the influence of the impaired redox equilibrium on platelet hyper-reactivity.

It is currently recognized that HC induces phenotypic vascular changes consistent with oxidative and nitrosative stresses. Once formed, superoxide participates in a number of reactions, yielding various free radicals, such as hydrogen peroxide, peroxynitrite, or oxidized-LDL. In particular, LDL particles in their native form induce hypersensitivity of platelets to agonists resulting in increased aggregation and secretion responses whereas, after oxidation, they become independent platelet activators in stirred platelet suspensions [56,57]. In this study, T2DM, as compared with HC, showed lower LDL-C levels. However, increased LDL-C characterizes HPR+ T2DM, compared with HPR- T2DM subjects, and CEPI PFA-100 is significantly and inversely correlated with LDL-C in T2DM but not in HC subjects, thus supporting the hypothesis that a pro-oxidant milieu triggered by hyperglycemia may amplify platelet activation via specific oxidized-LDL receptors and promote hyporesponsiveness to aspirin [58]. Noteworthy, a significant inverse correlation was found between SOD activity and LDL-C in a regression analysis including both T2DM and HC subjects, thus suggesting that hyperlipidemia is commonly involved in diminishing aspirin responsiveness in poorly controlled diabetes subjects and HC patients [59].

Some limitations of the study have to be taken into consideration. Firstly, classification of HPR+ and HPR- and their relationship with biomarkers were based on a single platelet assay. One could argue that the conclusions drawn are highly dependent on the test used and sometimes results obtained from different assays are not in agreement. Secondly, subjects of this study were all on 100 mg/day aspirin. Whether higher doses or increasing the frequency of aspirin administration from once to twice daily could be beneficial on plasma SOD effects on platelet function remains to be investigated.

## 4. Materials and Methods

### 4.1. Study Population

In this observational study, the potential population was initially represented by a total of 178 patients all on aspirin (100 mg/d), including patients affected by T2DM (*n* = 113) and patients with primary HC (*n* = 65). Patients were consecutively enrolled and diagnosis of T2DM was in accordance with the American Diabetes Association definition [60], whereas diagnosis of primary HC was in accordance with the Adult Treatment Panel (ATP) III criteria [61]. Exclusion criteria were concurrent therapy interfering with platelet function (i.e., nonsteroidal anti-inflammatory drugs, anticoagulants, P2Y12 inhibitors, COX-2 inhibitors), acute coronary syndrome or coronary revascularization procedures in the previous 12 months, active infections, inflammatory diseases, known platelet dysfunction or thrombocytopenia (< 100 x 10^9^ platelets/l). The study was approved by the Ethics Committee of the San Luigi Gonzaga Hospital (Project identification code: 213/INT, 3 June 2008). Written informed consent was obtained from all participants

### 4.2. Laboratory Measurements

Blood and urine samples for the determination of the biochemical parameters and platelet assays were collected after at least a 12 h overnight fast between 8:00 and 9:00 A.M., 18 h after the ingestion of the last aspirin dose for the determination of the investigated biochemical parameters and platelet assays. HbA1c was measured by automated high-performance liquid chromatography (HPLC) and expressed as percentage. Blood glucose levels were evaluated by glucose oxidase method. Standard laboratory techniques were used to determine TC, HDL-C, triglycerides (TG), and performed by the central laboratory of our Hospital. LDL-C levels were estimated with the Friedewald formula. For urinary metabolites, 24-h urine was collected and samples were added with the antioxidant 4-hydroxy-Tempo (1mmol/l) (Sigma Chemical Co., St. Louis, MO) and stored at −80 °C until use. Von Willebrand Factor (VWF) antigen concentrations were measured in citrated human plasma by an enzyme-linked immunosorbent assay (ELISA) (Corgenix Inc., Broomfield, CO, USA) and expressed in relative percent relative to pooled normal plasma.

For platelet assays, blood was drawn into evacuated tubes containing 3.8% Na-citrate and processed within 2 h of collection. For the other circulating parameters, supernatants (serum or plasma) were stored at −80 °C until use.

### 4.3. Platelet Assays

#### 4.3.1. PFA-100

PFA-100 (Siemens Healthcare Diagnostic Products GmbH, Marburg, Germany) assay with CEPI cartridge was utilized to assess aspirin-sensitivity among patients. PFA-100 is a point-of-care device that by simulating the in vivo primary haemostasis, records the time up to a maximum of 300 s needed for a whole blood sample to form a platelet plug closing an aperture on a membrane coated with agonists. Furthermore, closure time in the presence of CADP cartridge was also performed in each subject to exclude platelet dysfunction not dependent on aspirin. We categorized HPR+ patients with CEPI PFA-100 closure times < 193 s (the manufacturer’s lower limit of the normal range for aspirin-free healthy controls).

#### 4.3.2. Platelet Aggregation

To perform LTA in platelet-rich plasma (PRP) as described by Born [62], whole blood samples were centrifuged by using the Platelet Function Centrifuge (BioData Corporation, Horsham, PA, USA), designed to provide a rapid separation of PRP by a centrifugation for 30 sec and of platelet-poor plasma by a further centrifugation for 120 sec. Platelet aggregations in PRP were carried out using an eight-channel aggregation system (Platelet Aggregation Profiler, Model PAP-8, BioData Corporation), recorded for 5 min after agonist addition and expressed as % of maximal light transmittance. Platelets were stimulated by AA (0.5 mg/l) (Sigma, St. Louis, MO, USA), ADP (10 µmol/l) (Sigma, St. Louis, MO, USA), and collagen (4 mg/l) (Mascia Brunelli, Milan, Italy). Taking into account the cut-off values most frequently used in the past and associated with increased risk of suffering from adverse cardiac events despite daily aspirin intake, subjects in this study were considered HPR+ in the presence of LTA-AA > 20%, LTA-collagen > 60%, and LTA-ADP > 70% [63,64,65,66].

#### 4.3.3. Thromboxane Metabolites

Platelet maximal COX-1-dependent biosynthetical capacity was evaluated by measuring serum TXB_2_ concentrations by ELISA kit (Cayman Chemicals, Ann Arbor, MI, USA). The detection limit of the assay was 0.2 ng/mL.

The systemic endogenous production of TXB_2_ was evaluated by measuring urinary 11-dhTXB_2_ concentrations by ELISA kit (AspirinWorks Test kit, Corgenix, Inc., Broomfield, CO, USA) according to the manufacturer’s instructions and 11-dhTXB_2_ concentrations were normalized for urinary creatinine concentrations. The detection limit of the assay was 11 pg/mL.

### 4.4. Biochemical Parameters

#### 4.4.1. Inflammation, Endothelial Dysfunction and in Vivo Platelet Activation

Circulating levels of the following parameters were measured—i) IL-6, as marker of subclinical inflammation; ii) sRAGE, as antiatherogenic marker; iii) sE-selectin and sICAM, as markers of endothelial dysfunction; iv) sCD-40L and sP-selectin, as markers of in vivo platelet activation.

IL-6 and sCD40L concentrations were simultaneously quantified in the same plasma sample by Milliplex Map kit (Millipore Corporation, Burlington, MA, USA) based on the Luminex xMAP technology performing immunoassays on the surface of fluorescent-coated magnetic beads. Plasma concentrations of sRAGE, sICAM, sE-selectin, sP-selectin were measured by ELISA kits (Bender MedSystem, Vienna, Austria).

#### 4.4.2. Oxidative Stress

To evaluate the antioxidant potential to scavenge the main circulating ROS, plasma extracellular SOD (or SOD3) activity was measured by using a SOD assay kit (Cayman Chemical Company, Ann Arbor, MI, USA) and expressed as U/mL.

The measurement of the free radical-catalyzed lipid peroxidation product of AA 8-iso-PGF_2α_ is generally accepted as a reliable biomarker of oxidative stress [67,68]. The assay for the measurement of urinary levels of 8-iso-PGF_2α_ was performed by using a competitive ELISA kit (Oxford Biomedical Research, MI, USA) and 8-iso-PGF_2α_concentrations were normalized for urinary creatinine concentrations.

### 4.5. Statistical Analysis

All statistical analyses were performed using SPSS 26.0 for Windows (SPSS Institute, Chicago, IL). Values in the text and figures are expressed as mean ± SD or median, according to their distributions. Variable differences were tested by unpaired Student *t* test for normally distributed data or Mann–Whitney *U*-test for non-Gaussian data distribution. Differences in categorical variables were performed by using contingency tables and X^2^ or Fisher’s exact test. Results concerning PFA-100 were evaluated with the nonparametric Wilcoxon signed rank test since closure time is a harmonic variable with a maximum of 300 sec, which does not fluctuate proportionally within the scale. Pearson’s or Spearman correlation coefficients were used to examine the significance of correlation between variables, as appropriate. A multiple linear regression analysis, with variables significantly related to CEPI PFA-100 at univariate analysis, was performed to identify predictors of CEPI PFA-100. Results were considered significant for *p* < 0.05.

## 5. Conclusions

A comparable prevalence of high residual platelet reactivity was observed in T2DM and primary HC patients when the LTA-AA, LTA-collagen, urinary 11-dhTXB_2_ levels and CEPI PFA-100 were performed. A decreased activity of plasma extracellular SOD characterizes HPR+ subjects in both T2DM and HC and only SOD significantly predicted platelet reactivity according to platelet response to CEPI PFA-100. To conclude, in T2DM and HC, similarly, the impairment of redox equilibrium associated with a decrease of SOD activity could contribute to a suboptimal response to aspirin.

## Figures and Tables

**Table 1:** Clinical characteristics of primary hypercholesterolemia (HC) or Type 2 Diabetes Mellitus (T2DM) patients, n=164.

	HC (n = 61)	T2DM (n = 103)	p
Female gender, n (%)	28 (46)	50 (49)	0.87
Age (years)	63.9 ± 7.5	64.5 ± 7.0	0.58
BMI (kg/m^2^)	27.7 ± 3.2	29.3 ± 4.0	0.008
Fasting Glucose (mg/dl)	95.9 ± 10.1	159.5 ± 42	0.0001
HbA1c (%)	5.9 ± 0.33	7.99 ± 1.2	0.0001
TC (mg/dl)	194.2 ± 33.9	177.4 ± 37.7	0.005
HDL-C (mg/dl)	52.4 ± 10.4	43.8 ± 10.3	0.0001
TG (mg/dl)	126.1 ± 48.5	136.3 ± 66.2	0.29
LDL-C (mg/dl)	116.5 ± 32.4	103.2 ± 38.7	0.019
PLT (x10^3^/µl)	231 ± 42.3	239 ± 48	0.3
WBC(x10^3^/µl)	7.1 ± 1.6	7.1 ± 1.8	0.92
RBC(x10^3^/µl)	4.8 ± 0.44	4.7 ± 0.50	0.5
HCT (%)	41.9 ± 2.9	42.5 ± 3.9	0.29
MPV (fl)	8.4 ± 1.2	8.3 ± 0.9	0.53
vWF (%)	130.2 ± 56	123 ± 61	0.44
Creatinine	74.6 ± 20.3	75.3 ± 27.9	0.87
SBP (mmHg)	129.9 ± 10.6	134.3 ± 10.9	0.013
DBP (mmHg)	77.2 ± 7.0	76.4 ± 7.3	0.48
Previous cerebro-cardiovascular events, n (%)	28 (43)	26 (25)	0.62
Current smokers, n (%)	14 (23)	28 (27)	0.23
Medication use, n (%)			
Statins	45 (74)	70 (68)	0.006
Antihypertensives	28 (46)	81 (79)	0.0001
Insulin	0	24 (23)	N/A
Metformin	0	84 (82)	N/A
Any oral antidiabetic drugs	0	68 (66)	N/A

Data are presented as mean ± SD, otherwise specified. BMI: body mass index; HbA1c: glycosylated hemoglobin; TC: total cholesterol; HDL-C: high-density lipoprotein cholesterol; TG: triglycerides; LDL-C: low-density lipoprotein cholesterol; WBC: white blood cells; RBC: red blood cells; HCT: hematocrit; MPV: mean platelet volume; vWF: von Willebrand Factor; SBP: systolic blood pressure; DBP: diastolic blood pressure; N/A: not applicable.

**Table 2:** Clinical characteristics of primary hypercholesterolemia (HC) or Type 2 Diabetes Mellitus (T2DM) patients stratified on the basis of presence (+) or absence (-) of high on-aspirin platelet reactivity (HPR).

	HC			T2DM		
	HPR- (n=46)	HPR+ (n=15)	p	HPR- (n=79)	HPR+ (n=24)	p
Female gender, n (%)	20 (43)	8 (53)	0.78	40 (51)	10 (42)	0.63
Age (years)	63.9 ± 7.2	63.8 ± 8.5	0.96	65 ± 7.1	63 ± 6.7	0.21
BMI (kg/m^2^)	27.7 ± 3.4	27.5 ± 2.6	0.79	30 ± 4	28.5 ± 2.9	0.16
Fasting Glucose (mg/dl)	96.2 ± 11	94.9 ± 7.9	0.65	159 ± 38	162.3 ± 54	0.81
HbA1c (%)	5.9 ± 0.4	5.8 ± 0.24	0.26	8 ± 1.1	7.9 ± 1.4	0.62
TC (mg/dl)	193 ± 36.3	197.5 ± 26.2	0.66	172 ± 37	196 ± 34	0.005
HDL-C (mg/dl)	51.7 ± 10.2	54.7 ± 11.2	0.32	44 ± 11	42 ± 9	0.27
TG (mg/dl)	130 ± 51	114.1 ± 39.6	0.27	134 ± 68	144 ± 60	0.52
LDL-C (mg/dl)	115.4 ± 35	120 ± 23.4	0.63	99 ± 36	120 ± 43	0.013
PLT (x10^3^/µl)	228 ± 42	238.3 ± 42.8	0.45	241 ± 51	229 ± 36	0.2
WBC(x10^3^/µl)	7.09 ± 1.4	7.2 ± 2.1	0.92	7.13 ± 1.9	6.9 ± 1.6	0.58
RBC(x10^3^/µl)	4.8 ± 0.5	4.9 ± 0.41	0.48	4.7 ± 0.5	4.8 ± 0.5	0.71
HCT (%)	41.4 ± 2.4	43.4 ± 3.6	0.063	42 ± 3.7	44 ± 4	0.17
MPV (fl)	8.4 ± 1.1	8.6 ± 1.4	0.51	8.3 ± 0.9	8.6 ± 1	0.22
vWF (%)	127 ± 59	141.6 ± 50	0.37	122 ± 58	126 ± 68	0.78
SBP (mmHg)	130.1 ± 10.6	129.3 ± 11	0.8	135 ± 10	133 ± 13	0.42
DBP (mmHg)	77.4 ± 7.1	76.7 ± 7	0.73	77.2 ± 7.4	76 ± 6.5	0.65

Data are presented as mean ± SD, otherwise specified. BMI: body mass index; HbA1c: glycosylated hemoglobin; TC: total cholesterol; HDL-C: high-density lipoprotein cholesterol; TG: triglycerides; LDL-C: low-density lipoprotein cholesterol; WBC: white blood cells; RBC: red blood cells; HCT: hematocrit; MPV: mean platelet volume; vWF: von Willebrand Factor; SBP: systolic blood pressure; DBP: diastolic blood pressure.

**Table 3:** Biomarkers of oxidative stress, endothelial dysfunction, platelet activation, atherogenesis, and inflammation in primary hypercholesterolemia (HC) or Type 2 Diabetes Mellitus (T2DM) patients, n=164.

	HC (n = 61)	T2DM (n = 103)	p
SOD (U/mL)	0.23 ± 0.05	0.22 ± 0.05	0.4
8-iso-PGF_2__**α**_ (pmol/mmol creat)	141 ± 76	184 ± 126	0.016
sE-Selectin (pg/mL)	38.3 ± 21	48 ± 36	0.1
sICAM (ng/mL)	447 ± 61	457 ± 48	0.27
sP-Selectin (pg/mL)	174 ± 96	187 ± 62	0.298
sCD40L (pg/mL)	3.4 ± 4.2	12 ± 9.3	0.0001
IL-6 (pg/mL)	7.5 ± 4	9.7 ± 6	0.016
sRAGE (ng/mL)	1111 ± 640	826 ± 327	0.0001
TXB_2_ (ng/mL)	4.03 ± 2.3	4.4 ± 2.9	0.381
11-dhTXB_2_ (pg/mg creat)	1388 ± 591	1713 ± 1454	0.049

Data are presented as mean ± SD. SOD: superoxide dismutase; 8-iso-PGF2**α**: 8-iso-prostaglandin F2**α**; IL-6: interleukin-6; sRAGE: soluble Receptor of Advanced Glycation End Products; TXB2: thromboxane B2; 11-dhTXB2: 11-dehydro-thromboxane B2.

**Table 4:** Biomarkers of oxidative stress, endothelial dysfunction, platelet activation, atherogenesis, and inflammation in primary hypercholesterolemia (HC) or Type 2 Diabetes Mellitus (T2DM) patients, stratified on the basis of presence (+) or absence (-) of high on-aspirin platelet reactivity (HPR).

	HC			T2DM		
	HPR-	HPR+	p	HPR-	HPR+	p
SOD (U/mL)	0.24 ± 0.04	0.18 ± 0.06	0.002	0.23 ± 0.05	0.18 ± 0.04	0.0001
8-iso-PGF_2__**α**_ (pmol/mmol Creat)	140.1 ± 81	142 ± 61	0.92	161 ± 132	259 ± 63	0.0001
sE-Selectin (pg/mL)	37.7 ± 23	39.7 ± 13.5	0.73	46.1 ± 35	53.6 ± 37	0.39
sICAM (ng/mL)	450 ± 58	440 ± 69.3	0.64	457.02 ± 52	459 ± 36	0.89
sP-Selectin (pg/mL)	155 ± 91	245 ± 79.3	0.001	184 ± 63	196 ± 56	0.41
sCD40L (pg/mL)	2.8 ± 2.1	5.5 ± 7.7	0.052	10.8 ± 9.2	15.7 ± 9	0.003
IL-6 (pg/mL)	7.5 ± 4.6	7.5 ± 3.2	0.95	9.5 ± 6.1	10.2 ± 6.1	0.63
sRAGE (ng/mL)	1225 ± 694	807 ± 324	0.004	841 ± 355	777 ± 214	0.41
TXB_2_ (ng/mL)	3.8 ± 2.3	4.85 ± 2.2	0.12	4.1 ± 2.9	5.4 ± 2.6	0.59
11-dhTXB_2_ (pg/mg creat)	1270 ± 472	1753 ± 770	0.037	1550 ± 1270	2230 ± 1675	0.042

Data are presented as mean ± SD. SOD: superoxide dismutase; 8-iso-PGF2**α**: 8-iso-prostaglandin F2**α**; IL-6: interleukin-6; sRAGE: soluble Receptor of Advanced Glycation End Products; TXB2: thromboxane B2; 11-dhTXB2: 11-dehydro-thromboxane B2.
